# Nitrate or ammonium: Influences of nitrogen source on the physiology of a green alga

**DOI:** 10.1002/ece3.4790

**Published:** 2019-01-10

**Authors:** Sabrina C. Lachmann, Tabea Mettler‐Altmann, Alexander Wacker, Elly Spijkerman

**Affiliations:** ^1^ Institute of Biochemistry and Biology University of Potsdam Potsdam Germany; ^2^ Cluster of Excellence on Plant Sciences and Institute of Plant Biochemistry Heinrich‐Heine University Düsseldorf Germany; ^3^ Heisenberg‐Group: Theoretical Aquatic Ecology and Ecophysiology, Institute of Biochemistry and Biology University of Potsdam Potsdam Germany

**Keywords:** amino acids, carbon uptake kinetics, CO_2_ conditions, nitrogen, phosphorus limitation

## Abstract

In freshwaters, algal species are exposed to different inorganic nitrogen (N_i_) sources whose incorporation varies in biochemical energy demand. We hypothesized that due to the lesser energy requirement of ammonium (${\text{NH}}_{4}^{+}$)‐use, in contrast to nitrate (${\text{NO}}_{3}^{-}$)‐use, more energy remains for other metabolic processes, especially under CO_2_‐ and phosphorus (P_i_) limiting conditions. Therefore, we tested differences in cell characteristics of the green alga *Chlamydomonas acidophila* grown on ${\text{NH}}_{4}^{+}$ or ${\text{NO}}_{3}^{-}$ under covariation of CO_2_ and P_i_‐supply in order to determine limitations, in a full‐factorial design. As expected, results revealed higher carbon fixation rates for ${\text{NH}}_{4}^{+}$‐grown cells compared to growth with ${\text{NO}}_{3}^{-}$ under low CO_2_ conditions. ${\text{NO}}_{3}^{-}$‐grown cells accumulated more of the nine analyzed amino acids, especially under P_i_‐limited conditions, compared to cells provided with ${\text{NH}}_{4}^{+}$. This is probably due to a slower protein synthesis in cells provided with ${\text{NO}}_{3}^{-}$. In contrast to our expectations, compared to ${\text{NH}}_{4}^{+}$‐grown cells ${\text{NO}}_{3}^{-}$‐grown cells had higher photosynthetic efficiency under P_i_‐limitation. In conclusion, growth on the N_i_‐source ${\text{NH}}_{4}^{+}$ did not result in a clearly enhanced C_i_‐assimilation, as it was highly dependent on P_i_ and CO_2_ conditions (replete or limited). Results are potentially connected to the fact that *C. acidophila* is able to use only CO_2_ as its inorganic carbon (C_i_) source.

## 1 INTRODUCTION

Green algae (Chlorophyta), with around 6,500 recognized species (Guiry & Guiry, 2017), can be found in highly diverse habitats, such as in soils, streams, lakes, and even on stones, trees, and animals (Andersen, 1992). In these systems, algae might be exposed to different nutrient sources and limitations. Nitrogen is, next to phosphorus, an important nutrient that is more often (co‐)limiting in freshwater systems than previously thought (Elser et al., 2007). The assimilation of nitrogen into amino acids and proteins requires energy and organic carbon skeletons (Huppe & Turpin, 1994). Therefore, it is quite obvious that interactions between photosynthesis‐related processes and the acquisition of nitrogen occur, which is, for example, shown by a causal relationship between the assimilation of ${\text{NH}}_{4}^{+}$ and dark C_i_‐fixation under anaplerosis (Giordano, Norici, Forssen, Eriksson, & Raven, 2003; Vanlerberghe, Schuller, Smith, & Turpin, 1990) as well as photosynthetic CO_2_‐fixation (Turpin, Vanlerberghe, Amory, & Guy, 1991). In *Synechococcus* sp., the use of ${\text{NH}}_{4}^{+}$ over ${\text{NO}}_{3}^{-}$ stimulated photosynthesis and growth under a light limitation (Ruan & Giordano, 2017).

Most phytoplankton species are able to use ${\text{NH}}_{4}^{+}$ and ${\text{NO}}_{3}^{-}$ as the nitrogen source (Raven & Giordano, 2016). Several studies observed a higher photosynthetic or growth rate of macro‐ and microalgae for a certain N_i_‐source; reflected, for example, by different affinities to ${\text{NH}}_{4}^{+}$ and ${\text{NO}}_{3}^{-}$ uptake, or changes in a broad range of physiological parameters related to the growth response to different nitrogen sources (Ale, Mikkelsen, & Meyer, 2011; Beamud, Diaz, & Pedrozo, 2010; Giordano, 1997; Giordano & Bowes, 1997; Reay, Nedwell, Priddle, & Ellis‐Evans, 1999). For *Chlamydomonas* species, ${\text{NH}}_{4}^{+}$ is considered the preferred N_i_‐source, and negatively signals ${\text{NO}}_{3}^{-}$ assimilation (Fernandez & Galvan, 2007). These preferences might be a factor for competition, as the concentration of both nutrients varies through out the year in lakes (Kolzau et al., 2014). In contrast to ${\text{NO}}_{3}^{-}$, ${\text{NH}}_{4}^{+}$ is directly incorporated into amino acids by condensation with glutamate to form glutamine catalyzed by glutamine synthetase (Miflin & Lea, 1980; Sanz‐Luque, Chamizo‐Ampudia, Llamas, Galvan, & Fernandez, 2015). The demand to acquire CO_2_ increases with increasing nitrogen assimilation. In order to satisfy this demand, the provision of carbon skeletons via photosynthesis is partly energized from mitochondrial respiration (Giordano et al., 2003; Weger, Birch, Elrifi, & Turpin, 1988). Thus, phosphoenolpyruvate carboxylase (PEPc) activity (Giordano et al., 2003) is also enhanced.

Some phytoplankton species seem to grow faster with ${\text{NO}}_{3}^{-}$ than ${\text{NH}}_{4}^{+}$ (Dortch, 1990), potentially as an adaptation to the more available nitrogen source in their natural environment. Using ammonium, the algal cell avoids energy consuming steps of nitrogen reduction and the production of nitrate reductase (NR) and nitrite reductase (NiR) (Pritchard, Hurd, Beardall, & Hepburn, 2015; Raven, 1985). As no nitrification occurs in acidic lakes below pH 3 (Jeschke, Falagan, Knöller, Schultze, & Koschorreck, 2013), a high concentration of ${\text{NH}}_{4}^{+}$ (compared to ${\text{NO}}_{3}^{-}$) was observed in many of these lakes (Bissinger, Jander, & Tittel, 2000; Jeschke et al., 2013). Therefore, we suggest a preference for ammonium, and possibly a lack of NR activity, as an adaptation of acidophilic algae to their environment and decided to study the influence of N_i_‐source on several parameters describing the inorganic carbon acquisition (C_i_‐acquisition) of *Chlamydomonas acidophila *Negoro (SAG 2045). The strain was isolated from an acidic mining lake with a pH of about 2.7 (Gerloff‐Elias, Spijkerman, & Pröschold, 2005) and replete N_i_ concentrations (i.e. 0.22 mM), 91% of which is in the form of ${\text{NH}}_{4}^{+}$ (Bissinger et al., 2000).

We hypothesize that the acquisition of ${\text{NH}}_{4}^{+}$ compared to ${\text{NO}}_{3}^{-}$ allows a higher rate of nitrogen assimilation and that the reduced metabolic energy requirement of ${\text{NO}}_{3}^{-}$ use enables energy allocation to other cellular processes, such as photosynthesis and growth. Thus, C_i_‐uptake might be increased and photosynthetic parameters optimized to enhance photosynthesis, which probably also affects the cellular amino acid content. Some amino acids such as glutamate might be accumulated in ${\text{NO}}_{3}^{-}$‐grown cells as the protein synthesis is slowed and consequently the realized cell number decreased. Phosphorus also plays an important role in the energy budget as limiting P_i_‐conditions reduce the total adenylate concentration (Gauthier & Turpin, 1994; Theodorou, Elrifi, Turpin, & Plaxton, 1991) and consequently decreased, for example, the maximal photosynthetic and growth rate of *C. acidophila* (Spijkerman, 2010), and decreased the affinity for C_i_‐uptake in *Chlorella emersonii* (Beardall, Roberts, & Raven, 2005). The concentration of CO_2_ is the key factor for the activation of carbon‐concentrating mechanisms (CCMs); therefore, effects due to energy consuming CCMs (Raven & Beardall, 2014) might be amplified under low CO_2_. We realized that effects might be small as there are possibly no loss processes in the CCMs (Raven, Beardall, & Giordano, 2014).

It was previously shown that the influence of the N_i_‐source on different physiological parameters might depend on other factors, such as light (Ruan & Giordano, 2017) and CO_2_ (Giordano, 1997), but to our knowledge there are no studies combining two factors involved in energy and carbon metabolism in comparison with the effect of a different N_i_‐source. Therefore, as both C_i_ (Tittel, Bissinger, Gaedke, & Kamjunke, 2005) and P_i_ (Spijkerman 2008) have been identified as potential (co‐)limiting factors for *C. acidophila* in the acidic Lake 111 (Spijkerman, Stojkovic, Holland, Lachmann, & Beardall, 2016), we included both factors in our setup. Consequently, we studied interactions among P_i_‐ and CO_2_‐ supply/limitation under two different N_i_‐sources, in a full‐factorial design (eight different treatments). We expect the highest photosynthetic and C_i_‐assimilation rates at ${\text{NH}}_{4}^{+}$, P_i_‐ and CO_2_‐replete conditions, and the lowest at ${\text{NO}}_{3}^{-}$, P_i_‐, and CO_2_‐limiting conditions. The influence of provided N_i_‐sources on the ecophysiology of *C. acidophila* was examined by measuring a wide range of physiological parameters such as C_i_‐uptake kinetics, amino acid levels, and NR activity.

## 2 MATERIALS AND METHODS

### 2.1 Cultivation and number of cells

Three replicates of *C. acidophila* Negoro (SAG 2045) were cultivated semi‐continuously by daily dilution at a low steady‐state growth rate of 0.2/day in a climate chamber, to obtain stringent nutrient limiting conditions. Cultures were exposed to saturating light conditions (approximately 100 µmol photons m^–2^ s^–1^ as measured inside the culture flasks; Gerloff‐Elias, Spijkerman, & Schubert, 2005) at 20 ± 1°C in a modified Woods Hole Medium (Nichols, 1973); without silicate, at pH 2.5, buffered with FeCl_3_. We varied the N_i_‐source (${\text{NO}}_{3}^{-}$ or ${\text{NH}}_{4}^{+}$), P_i _concentration (P_i_‐limited: 1 µM and P_i_‐replete: 100 µM) and C_i_‐supply (low CO_2_: air, high CO_2_: 4.5%) in a full‐factorial design. Nitrogen was intended not to become a limiting nutrient, and therefore, ${\text{NO}}_{3}^{-}$ was provided in excess of 2 mM and ${\text{NH}}_{4}^{+}$ in excess of 1 mM. We added less ${\text{NH}}_{4}^{+}$ than ${\text{NO}}_{3}^{-}$ to prevent ammonium from causing an uncoupling of the photosynthetic H^+^ gradient (Krause & Behrend, 1986). All cultures were aerated with normal air or with CO_2_ enriched air from a gas cylinder (4.5% CO_2_ in air (v/v), Air Liquide) and each comprised 600 ml of culture volume within a 1‐l Erlenmeyer flask. Measured concentrations of CO_2_ in the flasks were 800 µM at high CO_2_, 14 µM at low CO_2_, P_i_‐limiting, and 3 µM at low CO_2_, P_i_‐replete conditions (following Spijkerman, Castro, & Gaedke, 2011). Algal growth was monitored by daily dilution and measurements of the optical density (OD) at 800 nm on a spectrophotometer (UV‐2401 PC; Shimadzu, Kyoto, Japan). Experiments were performed with algae in steady state, which means that the OD was stable for at least 20 days (three total exchanges of culture volume). The number of cells was measured by fixation with Lugol's iodine (1%) and counting on an automatic cell counter (CASY^®^1 TT, Schärfe System, Reutlingen, Germany).

### 2.2 Nutrient‐induced fluorescence transients

**Figure 1:**
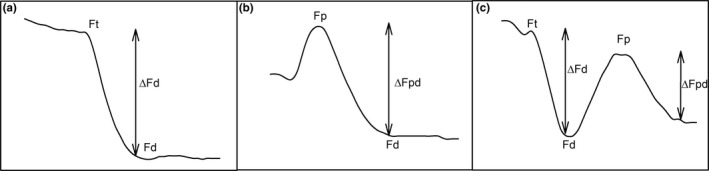
Possible shapes of nutrient‐induced fluorescence transients (NIFT) in response to the in vivo fluorescence of chl *a* and parameters for calculating the response. The parameters and shapes of the response are described in the text

Nutrient‐induced fluorescence transients (NIFTs) are a quick method to indicate nutrient depletion in algae (C_i_, N_i_, P_i_) due to transient change in chlorophyll *a* (chl *a*) fluorescence after a nutrient spike (Shelly, Holland, & Beardall, 2010; Spijkerman et al., 2016). On a Phyto‐PAM fluorometer (Heinz Walz GmbH, Effeltrich, Germany), the fluorescence (*F*_t_, chl *a* fluorescence in steady state and under actinic light) was recorded (Phytowin_v1.47) every three seconds without a saturating pulse for at least one minute (Gain: 8–18, actinic light: 120 µmol m^–2^ s^–1^). Subsequently, the response to an addition of P_i _(final concentration in cuvette: 10 µM KH_2_PO_4_), N_i_ (100 µM N_i_ as (NH_4_)_2_SO_4_ or NaNO_3_) or C_i_ (100 µM NaHCO_3_), and combinations of the nutrients, was recorded until the fluorescence signal remained constant again (*F*_d_). All spike solutions were prepared in acidified water of pH 2.5 (with the C_i_ solution diluted 1 min before addition of CO_2_ after conversion of most of the HCO_3_), and 20 µl was pipetted into a cuvette with 2 ml of diluted culture, to reach the previously denoted nutrient concentrations in the cuvette. Final P_i_ concentration was above 4 µM, based on results in Roberts, Shelly, and Beardall (2008), and N_i_ concentrations for maximal NIFT responses and a final CO_2_ concentration of 100 µM were selected, according to Young and Beardall (2003) and Spijkerman et al. (2016), respectively. A fresh sample was taken for each measurement to avoid potential influences of previous additions of nutrients. After addition of P_i_ and C_i_, a rapid decrease in the fluorescence was observed in P_i_‐ or CO_2_‐limited cultures. We calculated the Δ*F*_d_ (difference between lowest fluorescence after decrease, *F*_d_, and steady‐state fluorescence, *F*_t_ (Shelly et al., 2010)) as a percentage of F_t_ for these two nutrient additions (Figure 1a). The response of N_i_‐limited cells to ${\text{NH}}_{4}^{+}$ and ${\text{NO}}_{3}^{-}$ might be different, as described by Beardall, Young, and Roberts (2001), because we found a small rise of fluorescence (*F*_p_, highest fluorescence as response to nutrient) followed by a drop to *F*_d_ after ${\text{NH}}_{4}^{+}$ addition, and a strong rise of fluorescence to *F*_p_ with a recovery to initial values after ${\text{NO}}_{3}^{-}$ injection (*F*_t_). Therefore, for these two nutrients, we calculated Δ*F*_pd_ as representing the difference between the highest fluorescence at the top of the peak and the lowest value after decreasing (Figure 1b). Under colimitation of three nutrients, a third response was observed: after adding nutrient combinations (P_i_ and C_i_, C_i_ and ${\text{NH}}_{4}^{+}$, or all three nutrients) to the culture, at first a decrease (*F*_d_), then an increase (*F*_p_), and finally a second decrease of the fluorescence was observed (second *F*_d_). We calculated the response by forming the sum of Δ*F*_d _and Δ*F*_pd_ (Figure 1c). Such a response to two potentially limiting nutrients has been shown before for *C. acidophila* growing under P_i_‐ and C_i_‐deplete conditions (Spijkerman et al., 2016).

### 2.3 Traditional nutrient enrichments

We tested for the growth limiting nutrient of cultures by performing enrichment experiments. Eighty microliters of spike solutions was added (P_i_: 1 mM; N_i_ (${\text{NO}}_{3}^{-}$ or ${\text{NH}}_{4}^{+}$ depending on culture conditions) and C_i_: both 10 mM) to 8 ml of culture. Acidified water was added as a control. After three days of growth under the above‐mentioned culture conditions, the optical densities were measured. The influence of nutrients was detected by calculating differences in biomass yield between enriched and control cultures.

### 2.4 Chl *a*, protein content, and NR activity

During steady state, part of the culture, remaining after daily dilution, was centrifuged (2,000 ***g***, 5 min, 6°C), washed with demineralized water and quickly frozen at −80°C. For extraction and following analyses, these pellets of algae were resuspended in 0.7 ml extraction buffer (pH 8), which consisted of 50 mM HEPES (N‐2‐Hydroxyethyl piperazine‐N‐2‐ethane sulfonic acid), 0.1% Triton‐X‐100, 10% glycerol and 1 mM Na_2_EDTA (Ethylenediaminetetraacetic acid), DTT (Dichlorodiphenyltrichloroethane; 1 mM), and protease inhibitor (S8820, Sigma‐Aldrich, 18 ng/ml). Glass beads (1.0 mm, BioSpec Products, Inc., Bartlesville, USA) were added to homogenize cells in a cooled bead beater (Precellys 24; PEQLAB, Erlangen, Germany) by shaking 6 times for 10 s at 5,000 rpm. After centrifugation (5 min, 1,800 ***g***; Biofuge Stratos; Heraeus, Hanau Germany), the supernatant was enriched with 10 mM MgCl_2_ (final concentration) and stored at −20°C for <1 week, until analysis.

The NR activity was standardized for protein, and this was measured by a method that determines chl *a* and protein in the same sample (Peterson, 1977). To measure chl *a* concentration in cells, cell extracts were well‐mixed with precooled acetone (90%) in a bead beater (3 × 10 s at 5,000 rpm) and centrifuged (5 min at 18,000 ***g*** at 7°C). We measured absorption of the supernatant at 750, 664 and 647 nm in a spectrophotometer, and calculated concentrations of chl *a* according to Jeffrey and Humphrey (1975). The remaining pellet was used for protein determination. For the measurement of protein content, pellets were resuspended in 500 µl of SDS (1%) in NaOH (0.1 M), following Peterson (1977). Bovine serum albumin (BSA) was used as a standard. Two dyes were freshly prepared containing, A: CTC (0.1% CuSO4 × 5 H_2_O, 0.2% NaKTartrate, 10% Na_2_CO_3 _(w/v)), 10% SDS, 0.8 M NaOH and H_2_O mixed in equal amounts, and B: Folin reagent diluted in a fivefold amount of water. Firstly, to the resuspended pellet or standard 500 µl of dye A was added and directly mixed. After 10 min, 250 µl of dye B was pipetted in and mixed. Another 30 min of incubation was needed before 300 µl aliquots was filled in wells of a 96‐well microplate and absorption was measured at 750 nm (infinite F200PRO, TECAN, Männedorf, Switzerland).

We modified the NR activity measurements following Chen, He, and Hu (2012), as follows. Fifty µl of cell extracts was mixed with reaction solution consisting of 50 mM HEPES (pH 7.5), 10 mM KNO_3_, and 0.1 mM EDTA. The reaction was started by adding 5 µl NADH (40 mM), while extracts incubated in a 30°C water bath. The incubation time varied based on potential activities (15–30 min; which was linear and tested beforehand) and the enzyme reaction was stopped by adding 50 µl zinc acetate solution (1 M). Afterward, extracts were centrifuged for 3 min at 6,700*** g*** (Minispin; Eppendorf; Hamburg, Germany) and nitrite content in the supernatant was determined with the help of two coloring solutions. These were mixed 1:1 (10 g/L sulfanilamide in 3 N HCl and 200 mg/L N‐(1‐Naphthyl)ethylenediamine dihydrochloride in H_2_O) directly before use, after which, 100 µl of the coloring mixture was added to 100 µl supernatant in an 96‐well microplate. The filled microplates were covered with a plastic film and incubated for 15 min under gentle shaking. Finally, absorption was measured in a multimode microplate reader at 540 nm. This absorption was converted to NR activity via a calibration curve and presented as nmol nitrite min^‐1^ mg protein^‐1^.

### 2.5 C_i_‐accumulation factor

We measured the C_i_‐accumulation factor (CCF) to check for the effect of N_i_‐source on C_i_‐accumulation. Concentrated cell suspension (after centrifugation at 1,500 *g* for 5 min and resuspension in the growth medium, and that to approached an OD of 2) was illuminated for one hour to deplete C_i_ and then placed on a silicon oil layer on the top of a killing fluid (Spijkerman, 2005). The accumulation of C_i_ was measured following Badger, Kaplan, and Berry (1980) and Spijkerman, Stojkovic, and Beardall (2014). Preliminary experiments showed that an effective oil mixture of 1:1 or 1:2 (v/v) of silicon oil “type 3,” and “500” for gas chromatography (Merck, Darmstadt, Germany), sufficed. We provided between 50 and 70 µM NaH^14^CO_3_, with a specific activity of about 1,739 GBq/mmol (PerkinElmer, Germany), for the algae while illuminating with 200 µmol PAR m^‐2^ ^‐1^ (incident irradiance), and the carbon uptake was stopped after 10 s by centrifugation (12,000 ***g***, 15 s). Centrifuge tubes were flash frozen in liquid nitrogen, and the algal pellets removed simply by cutting off the end of the tube. The pellets were resuspended in 400 µl NaOH (0.1 M), and then, we transferred 150 µl into the same amount of NaOH (0.1 M; total fraction) or HCl (0.5 M in methanol; acid stable fraction). Acid‐labile carbon in the HCl samples evaporated under a fume head over night. 2.5 ml of Ultima Gold (PerkinElmer) was then added before counting in a liquid scintillation analyzer (Tri‐Carb 2,810; PerkinElmer). We calculated the C_i_‐accumulation from the difference between total and acid stable fractions. After 15‐ to 20‐min incubation with 37 kBq ^3^H‐H_2_O (specific activity: 37 kBq · mmol^‐1^ Hartmann‐analytic), cell volume was measured according to Beardall (1981). The CCF of the accumulated C_i_ over the 10 s was calculated in two ways: one based on cell volume determined with ^3^H‐H_2_O, and one, based on the cell volume calculated with cell diameter analyzed with the automatic cell counter CASY®1 TT (Schärfe System, Reutlingen, Germany).

### 2.6 ^14^C‐fixation rates

To check for the effect of N_i_‐source on the rate of C_i_‐acquisition, we measured the primary productivity by C_i_‐fixation rate. Each culture was sampled and measured as quickly as possible to avoid changes in the CO_2_ equilibration. Three technical replicates were kept in light while the one control was placed in the dark. We added a final concentration of 2 µM NaH^14^CO_3_ (3,480 Bq/ml) from stock solution (1.74 GBq/mmol specific activity; PerkinElmer, Germany) and incubated the algae depending on their P‐status ranging from 2 to 13 min. The fixation of C_i_ was stopped by rapid filtration over a 0.25‐µm nucleopore filter (Whatman, Maidstone, UK) under a maximal pressure of 200 mbar and rinsing with demineralized water. For detection of fixed particulate organic ^14^C (PO^14^C), filters were dissolved in 0.5 ml Soluene (Perkin Elmer). Separately, 0.5 ml of each sample was taken to measure the total activity of ^14^C (T^14^C). We added 2.5 ml of scintillation fluid (Ultima Gold, PerkinElmer) to all samples before counting radioactive decay in a liquid scintillation analyzer (Tri‐Carb 2810 TR, Perkin Elmer). The ^14^C fixation rate was calculated following equation 1; with DIC (the concentration of dissolved C_i_) measured by an injection of 4 ml culture into a liquid carbon analyzer (High TOC; Elementar Analysensysteme GmbH, Hanau, Germany), and 1.06, a factor for the isotope‐discrimination between ^12^C and ^14^C by phytoplankton (Steemann, 1952).

$${}^{14}\text{C fixation rate =}\;\frac{\left(\frac{PO^{14}C\left(\text{corr.}\right)}{\text{ml}(\text{filter})\times \text{incubation time}}\times \text{activity(spec}{\text{.)}}^{\;\text{- 1}}\right)\times \left(\frac{\text{DIC}}{T^{14}C}\right)\times 1.06}{\text{chlorophyll}\;a\;\text{concentration}}$$

### 2.7 Extraction of algal samples and measurements of amino acids (HPLC)

The accumulation of amino acids in cells can indicate hampering of protein synthesis. The effect of N_i_‐sources on the steady‐state levels of amino acids was therefore measured by extracting the amino acids from algal cells. Firstly, 10 ml of culture was quickly cooled down to −60°C in 20 ml quenching solution, consisting of 70% methanol (cooled by −70°C ethanol), flash frozen in liquid nitrogen, and stored at −80°C. Samples were dried and then extracted according to Mettler et al. (2014). Extracts were dissolved in 50 µl HCl (0.1 M). As the optimal pH for derivatization is between 8.2 and 10, we added 2 µl of 1 N NaOH and 8 µl H_2_O to 10 µl samples, from which 10 µl were taken for derivatization by AccQ‐Tag Ultra Reagent Powder (Waters Corporation, Milford, MA, USA), according to the manufacturer's instructions. Derivates were separated by liquid chromatography and detected at 260 nm using a 1,290 UHPLC system coupled to diode array detector (Agilent, USA), according to Rademacher et al. (2016).

### 2.8 C_i_‐uptake kinetics and photosynthetic measurement

To assess the effect of N_i_‐source on the efficiency for C_i_‐acquisition, we measured C_i_‐uptake kinetics. Algal cultures were centrifuged (5 min at 1,500 g) and resuspended in fresh medium (pH 2.5) without the limiting nutrient, in order to concentrate cells. ODs differed among varying P_i_ and CO_2_ (P_i_‐replete and low CO_2_: 0.20 – 0.23, P_i_‐replete and high CO_2_: 0.31 – 0.36, P_i_‐limited low CO_2_: 0.31 – 0.42; P_i_‐limited, high CO_2_: 0.44 – 0.67). Oxygen evolution rates were measured, following Lachmann, Maberly, and Spijkerman (2016b), with a Clark electrode at an incident saturating light intensity of 500 µmol m^‐2^ s^‐1^ for the dense samples in a light dispensation system (Illuminova, Uppsala, Sweden). The half‐saturation constant for C_i_ K_0.5_(C_i_) by photosynthesis, the maximal uptake rate *V*_max_, and the affinity *V*_max_/*K*_0.5_(C_i_) were calculated by modeling the response of oxygen evolution rates to C_i_ concentrations and performing a linearization, according to Hofstee (1952). As measurements were taken under pH 2.5, all C_i_ quickly converts to CO_2_ and therefore, hereafter, K_0.5_(C_i_) is mentioned as K_0.5_(CO_2_).

Additionally, photosynthetic electron transport rate was also measured via rapid light curves using a Phyto‐PAM fluorometer (Heinz Walz GmbH, Effeltrich, Germany) and following Grzesiuk, Wacker, and Spijkerman (2016). We determined the electron transport rate (ETR) with PhytoWin (V2.13) under different light intensities, and calculated alpha, the slope at the beginning of the light curve. This parameter represents the relative photosynthetic efficiency on the basis of electrons, which might be related to the usage of different nitrogen sources.

### 2.9 Chemical analyses

For determining the content of carbon and nitrogen (Supporting information Tables S2 and S3) in cells, a defined volume of algal cultures was filtered through precombusted (4 hr at 450°C) GF/F filters. Filters were dried at least 48 hr at 45°C (WTC binder, Tuttlingen, Germany), packed in tin cartridges (10x10 mm, HEKAtech GmbH, Wegeberg, Germany) and measured in a CHNS‐O Elemental Analyzer (EA 3000; EuroVector SpA, Milan, Italy).

Particulate phosphorus content of cells (Supporting information Tables S2 and S3) was determined by filtering algal culture on polysulphone filters (0.45 µm; Pall Corporation, Port Washington, NY, USA). Subsequently, filters were oxidized by adding K_2_S_2_O_8_ and autoclaved at 120°C and 120 kPa for 1 hr. The molybdate blue reduction method according to Murphy and Riley (1962) was used, and phosphorus concentrations were measured at 880 nm on a spectrophotometer (UV‐2401 PC; Shimadzu, Kyoto, Japan), and compared to a similarly treated calibration curve.

For cellular chl *a* content, concentrations were determined by filtering culture samples on glass fiber, GF/F filters (Whatman, Buckinghamshire, UK). Chl *a* was extracted overnight with 60°C warm ethanol (90%). Measurements of extracts were conducted in a fluorometer (TD‐700, Turner Designs, GAT Bremerhaven, Germany), following Welschmeyer (1994), and quantified using a calibration curve prepared from commercially obtained chl *a* (Sigma).

### 2.10 Statistical analyses

Statistical analyses were performed with the software R version 3.4 (R Core Team, 2017). Influences of nutrients on tested variables were detected using three‐way ANOVAs. To estimate the particular effects of the N_i_‐source, we calculated contrasts between the ${\text{NO}}_{3}^{-}$ and ${\text{NH}}_{4}^{+}$ treatments within each CO_2_ – P combination using the R package “emmeans” (Russell, 2018). As cutoff level of significance, *p* > 0.05 was used. This way, we test straightforwardly for the modulating effects of CO_2_ and P_i_ availability on the potential of N_i_‐source utilization.

## 3 RESULTS

The order of the results will follow the potential path of nitrogen through the metabolism of the alga. At first, the limitation(s) of cells is described to better understand the results. In short, we consider the NR activity as the first step of the nitrate metabolism and follow this by the synthesis of amino acids for both N_i_‐sources. Then, we examine the production of chlorophyll, whose content will influence parameters involved in photosynthesis. Finally, the cell density is presented, which reflects the total efficiency of all metabolic processes resulting in population productivity, as our experiments were performed in a fixed steady‐state rate of growth.

### 3.1 Detection of nutrient limitations

**Figure 2:**
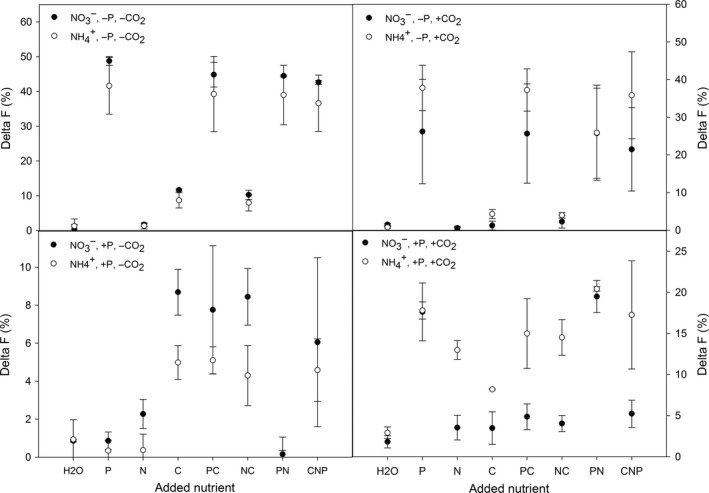
Nutrient‐induced fluorescence transients (NIFT) response to added nutrients in all culture treatments calculated differently as described in methods; data points reflect mean and standard deviation of three replicates. In each panel, responses are compared between algae provided with NH_4_^+^ or ${\text{NO}}_{3}^{-}$ under a certain nutrient condition; a: P_i_‐limited conditions under low CO_2_, b: P_i_‐limited conditions under high CO_2_, c: P_i_‐replete conditions under low CO_2_, d: P_i_‐replete conditions under high CO_2_. Please note the differences in scales on the y‐axes between panel a, b and c, d

We compared two different methods for verifying the suggested factors (co‐)limiting the differently treated cultures, as the identification of P_i_ and C_i_ limitations was essential for the basis of the conducted experiment. Traditional enrichment experiments detect limitations via additions of nutrients, after some days of growth, and reveal the growth limiting nutrient *in *sensu Liebig (Sperfeld, Raubenheimer, & Wacker, 2016; von Liebig, 1841). In contrast, nutrient‐induced fluorescence transients (NIFTs) can be used as a much more rapid method for detecting nutrient (co‐)limitations affecting photosynthesis and consequently metabolic rates (i.e., Blackman limitation). In these, the direct photosynthetic response to nutrient additions is visible immediately and reflects the current nutrient status (Spijkerman et al., 2016). Both the traditional enrichment (Supporting information Table S1) and NIFT experiments (Figure 2) confirmed the desired P_i_‐limitation. Additionally, NIFT experiments revealed further effects of nutrients on photosynthesis and possible interactions, for which traditional enrichment experiments were not sensitive enough. We detected a Δ*F*_d_ of around 10% if C_i_ was added to low CO_2_‐grown cells, implying a slight CO_2_ limitation in all these cultures (Figure 2a, c). When ${\text{NO}}_{3}^{-}$ was the available nitrogen source, under low CO_2_, the responses of cells to nutrient additions were slightly, but consistently, stronger than of cells grown with ${\text{NH}}_{4}^{+}$ (Δ*F*_pd_, Figure 2a, c). In contrast, the response of ${\text{NH}}_{4}^{+}$‐ grown cells was often stronger than ${\text{NO}}_{3}^{-}$‐grown cells under high CO_2_ conditions (Figure 2b, d). Against expectations, we found a clear response to all added nutrients (i.e., C, N, and P) in the ${\text{NH}}_{4}^{+}$/P_i_‐replete/highCO_2_ treatment (Figure 2d). The responses of nutrient combinations (sum of Δ*F*_d _and Δ*F*_pd_) included fast evolving transients as depicted in Figure 1c, suggesting the presence of a co‐limitation for all three nutrients. How these limitations relate to each other in a physiological sense could not be unraveled.

### 3.2 NR activity

**Figure 3:**
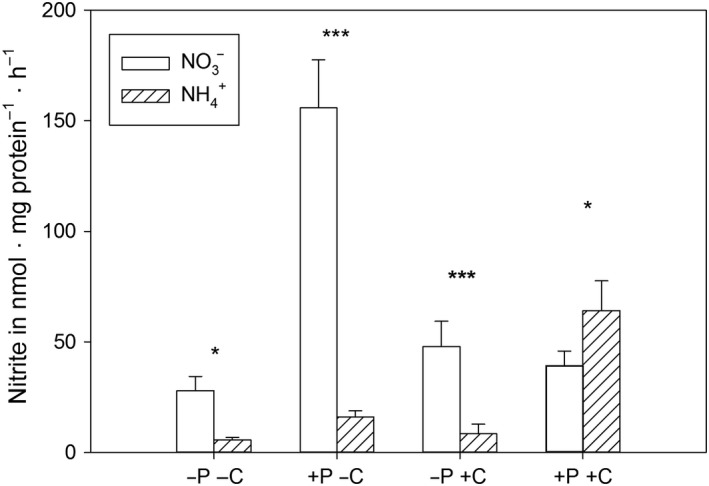
Mean and standard deviation of the activity of NR [nmol nitrite mg protein^–1^ hr^–1^] (*n* = 3). Asterisks show significant differences between treatments differing in nitrogen sources (contrast analysis, * *p* < 0.05, ***p < 0.001), letters and signs of the x‐axis describe treatments: ‐P: P_i_‐limitation, +P: P_i_‐replete, ‐C: low CO_2_, +C: high CO_2_

The activity of NR might be the first reaction to changing nitrogen conditions: and we hypothesized that the studied algae might lack NR activity as an adaptation to an acidic environment without ${\text{NO}}_{3}^{-}$. In contrast to this expectation, *C. acidophila* had NR activity and the N_i_‐source affected the activity of NR in all combinations of factors (Figure 3, three‐way ANOVA, N × P × CO_2_: *F*_1,16_ = 110.3, *p* < 0.001, Supporting information Table S4). In detail, by comparing the responses of NR between N_i_‐sources within each CO_2_ – P combination (Figure 3, contrast analyses, for details see methods), a higher activity was observed in treatments with ${\text{NO}}_{3}^{-}$ instead of ${\text{NH}}_{4}^{+}$ (contrast analyses, *p* < 0.05), except under P_i_‐replete and high CO_2_ conditions. Unexpectedly, in the latter case, a higher activity was detected when ${\text{NH}}_{4}^{+}$ was provided compared to ${\text{NO}}_{3}^{-}$ (contrast analysis, *p* < 0.05), which might be due to the N_i_‐limitation. The strongest positive effect of ${\text{NO}}_{3}^{-}$ on the NR activity was observed under low CO_2_ and P_i_‐replete conditions, with an eightfold increase compared with the ${\text{NH}}_{4}^{+}$ treatment (contrast analysis, *p* < 0.001). We found an interaction between N_i_ and both P_i_ and CO_2_ (three‐way ANOVA, N × P: *F*_1,16_ = 9.4 *p* < 0.01, N × CO_2_: *F*_1,16_ = 72.8, *p* < 0.001, Supporting information Table S4).

### 3.3 Amino acids

The condensation of ammonia and glutamate to glutamine is the first step of fixing nitrogen within the algal cell. Since it needs additional steps of reduction, we expected a slower turnover from nitrogen to amino acids when cells were grown with the more oxidized ${\text{NO}}_{3}^{-}$ compared to ${\text{NH}}_{4}^{+}$. This might be visible in different steady‐state levels of amino acids in cells grown under different N_i_‐sources. Glutamate generally accumulated in cells grown with ${\text{NO}}_{3}^{-}$, but surprisingly, ${\text{NO}}_{3}^{-}$ also led to an increase in threonine, alanine, and glycine under P_i_‐limited conditions, and on tyrosine under high CO_2_ conditions (Table 1, Supporting Information Table S7). The positive influence of ${\text{NO}}_{3}^{-}$ on the cellular content of valine was only observed under high CO_2_ conditions and limiting P_i_‐supply (Table 1, Supporting Information Table S7). Additional to our main interest concerning the influence of N_i_‐source, the major differences in amino acid levels were found between high and low phosphorus supply. Six of the nine analyzed amino acids were increased under P_i_‐limited conditions (alanine, glycine, isoleucine, leucine, threonine, valine; Table 1 and Supporting information Table S7). Furthermore, the level of glycine was also affected by the variation of CO_2_ (Supporting information Table S7).

**Table 1:** Cellular content of different amino acids (in pmol · 10^‐6^ cells) in *Chlamydomonas acidophila*, grouped by the factors influencing them

Amino acid	Low CO_2_				High CO_2_			
	P_i_‐limited		P_i_‐replete		P_i_‐limited		P_i_‐replete	
	${\text{NO}}_{3}^{-}$	${\text{NH}}_{4}^{+}$	${\text{NO}}_{3}^{-}$	${\text{NH}}_{4}^{+}$	${\text{NO}}_{3}^{-}$	${\text{NH}}_{4}^{+}$	${\text{NO}}_{3}^{-}$	${\text{NH}}_{4}^{+}$
Influence of N_i_‐source								
General influence								
Glutamate	639 (279)^a^	99.1 (9.3)^b^	361 (80)^a^	134 (119)^b^	300 (21)	98.5 (34.9)	364 (119)^a^	65.4 (46.1)^b^
Threonine	212 (16)^a^	94.4 (36.1)^b^	109 (36)	98.9 (36.3)	325 (23)^a^	121 (79)^b^	183 (53)^a^	39.4 (19.2)^b^
Influence under P_i_‐limitation								
Alanine	752 (125)^a^	247 (60)^b^	402 (53)	312 (84)	906 (76)^a^	210 (100)^b^	255 (85)	146 (84)
Glycine	1710 (393)^a^	500 (118)^b^	607 (140)	947 (310)	2,255 (5)^a^	423 (192)^b^	139 (65)	243 (40)
Influence under high CO_2_								
Tyrosine	214 (33)	125 (70)	186 (48)	110 (45)	295 (38)^a^	86.3 (40.8)^b^	222 (41)^a^	69.6 (66.1)^b^
Influence under high CO_2 _and P_i_‐limitation								
Valine	130 (109)	201 (94)	84.2 (20.8)	182 (53)	407 (148)^a^	117 (67)^b^	131 (53)	46.2 (29.4)
No Influence on N_i_‐source								
Isoleucine	155 (71)	129 (86)	84.2 (38.2)	165 (112)	207 (6)	186 (62)	66.2 (29.3)	33.6 (11.6)
Leucine	245 (117)	177 (156)	99.2 (44.4)	107 (89)	278 (11)	270 (76)	71.4 (20.7)	34.1 (27.4)
Phenylalanine	268 (151)	247 (220)	140 (56)	297 (360)	206 (30)	300 (249)	190 (71)	28.2 (17.1)

Notes

Mean and standard deviation in parentheses. Different letters in superscript indicate significant differences in cellular amino acid contents between the two N_i_‐sources within each P_i_ ‐ CO_2_ combination (three‐way ANOVA followed by contrast analyses with R package emmeans).

### 3.4 Chl *a* per cell

**Figure 4:**
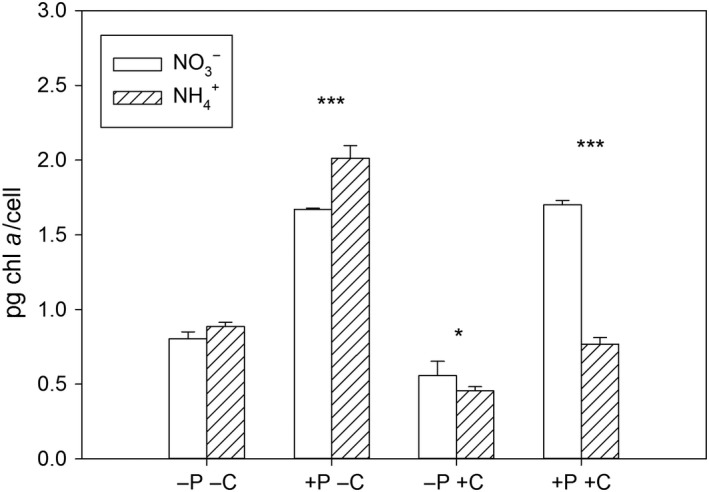
Mean and standard deviation of chl *a* content in *C. acidophila* (*n* = 3). Asterisks show differences between treatments differing in nitrogen sources (contrast analysis, *p < 0.05, ***p < 0.001), letters and signs of the x‐axis describe treatments: ‐P: P_i_‐limitation, +P: P_i_‐replete, ‐C: low CO_2_, +C: high CO_2_

Chl *a* is produced from glutamate and plays an important role for the photosynthetic capacity of an algal cell. Since the different nitrogen sources affect glutamate synthesis differently, we also expected changes in chl *a *levels. Indeed, the chl *a* content per cell was significantly affected by the N_i_‐source, but the effects of the N_i_‐source clearly changed with the supply of P_i_ and CO_2_ (three‐way ANOVA, N_i_ × P_i_ × CO_2_: *F*_1,16_ = 156, *p* < 0.001, Figure 4, Supporting information Table S4). The presence of ${\text{NH}}_{4}^{+}$, instead of ${\text{NO}}_{3}^{-}$, enhanced the chl *a* content per cell under P_i_‐replete and low CO_2_ conditions (contrast analysis, *p* < 0.001, Figure 4), but under high CO_2_, ${\text{NO}}_{3}^{-}$‐grown cells had a higher chl *a* content than ${\text{NH}}_{4}^{+}$‐grown cells (contrast analysis, *p* < 0.001, Figure 4).

### 3.5 ^14^C‐fixation rates

**Figure 5:**
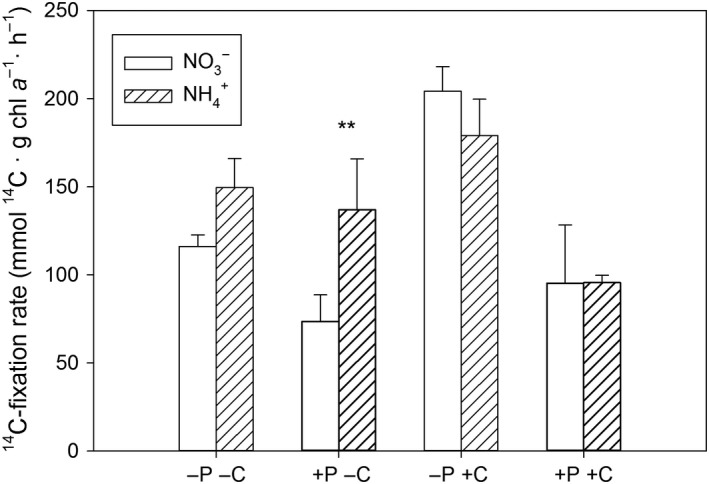
Mean and standard deviation of the ^14^C‐fixation rate [mmol ^14^C g chl *a* ^–1^ hr^–1^] (*n* = 3). Asterisks show differences between treatments differing in nitrogen sources (contrast analysis,** *p* < 0.01), letters and signs of the x‐axis describe treatments: ‐P: P_i_‐limitation, +P: P_i_‐replete, ‐C: low CO_2_, +C: high CO_2_

The C_i_‐fixation rate was measured by ^14^C‐labelled incorporation of C_i_, and these rates might be decreased under nutrient and energy limiting conditions. An interaction of the N_i_‐source with the CO_2_ supply was observed (three‐way ANOVA, N_i_ × CO_2_: *F*_1,16_ = 14.3, *p* < 0.01, Figure 5, Supporting information Table S4), but no higher or other interaction with P_i_ was discerned (Supporting information Table S4). The N_i_‐source influenced the ^14^C‐fixation rates only under low CO_2_ conditions; under these conditions, a higher rate was observed when ${\text{NH}}_{4}^{+}$ was used by algal cells instead of ${\text{NO}}_{3}^{-}$ (contrast analysis, *p* < 0.05).

### 3.6 C_i_‐uptake kinetics

Kinetic parameters of photosynthesis reflect the effectivity of C_i_‐acquisition: which we expected to be enhanced in ${\text{NH}}_{4}^{+}$‐grown cells compared to cells grown in the presence of ${\text{NO}}_{3}^{-}$. The kinetic parameter *V*_max_ was lower under P_i_‐limiting than P_i_‐replete conditions (three‐way ANOVA, P_i_: *F*_1,16_ = 15, *p* = 0.001, Supporting information Table S5). Among N_i_‐treatments, only under high CO_2_ and P_i_‐replete conditions was the *V*_max_ of ${\text{NH}}_{4}^{+}$‐provided cells lower than the value of ${\text{NO}}_{3}^{-}$‐cells (contrast analysis, *p* < 0.01, Table 2), which was probably due to the nitrogen limitation in the ${\text{NH}}_{4}^{+}$ treatment. Therefore, we suggest that *V*_max_ was unaffected by N_i_‐source, but negatively affected by nitrogen limitation.

**Table 2:** Mean and standard deviation (mentioned in parentheses) of CO_2_ uptake kinetics as measured by oxygen evolution (*n* = 3), *V*_max_ (mmol O_2_ (g chl *a* h)^–1^), K_0.5_ (CO_2_) (µM) and *V*_max_/*K*_0.5_

Parameter	Low CO_2_				High CO_2_			
	P_i_‐limited		P_i_‐replete		P_i_‐limited		P_i_‐replete	
	${\text{NO}}_{3}^{-}$	${\text{NH}}_{4}^{+}$	${\text{NO}}_{3}^{-}$	${\text{NH}}_{4}^{+}$	${\text{NO}}_{3}^{-}$	${\text{NH}}_{4}^{+}$	${\text{NO}}_{3}^{-}$	${\text{NH}}_{4}^{+}$
*V*_max_	48 (9)	56 (6)	73 (6)	64 (4)	42 (7)	33 (16)	64 (6)^a^	38 (14)^b^
*K*_0.5_ (CO_2_)	1.3 (0.6)	1.4 (0.2)	2.1 (0.4)	2.0 (0.4)	4.5 (1.0)	5.0 (0.8)	4.2 (0.9)^a^	2.1 (0.6)^b^
*V*_max_/*K*_0.5_	44 (27)	41 (4)	36 (5)	33 (5)	9 (1)	7 (4)	16 (2)	18 (5)

Notes

Different letters in superscript indicate significant differences in kinetic parameters between the two *N*‐sources within each P ‐ CO_2_ combination (three‐way ANOVA followed by contrast analyses with R package emmeans).

The N_i‐_source and the P_i_‐supply in concert with the CO_2_ conditions affected the half‐saturation constant *K*_0.5_ (CO_2_) (three‐way ANOVA, N_i_ × P_i_ × CO_2_: *F*_1,16_ = 5.1, *p* < 0.05, Table 2 and Supporting information Table S5). Within P_i_‐replete‐high CO_2_‐conditions, the *K*_0.5_ (CO_2_) was lower when ${\text{NH}}_{4}^{+}$ was the N_i_‐source provided (contrast analysis, *p* < 0.01, Table 2), indicating the onset of a slight C_i_‐limitation, also observed in the NIFT measurements (Figure 2d). As a result of the slight C_i_‐ and/or N_i_‐limitation present in the high CO_2_/P_i_‐replete treatment, some of the interactions became statistically significant.

A higher affinity for CO_2_ (*V*_max_/*K*_0.5_) was detected in low CO_2_ conditions (Table 2, three‐way‐ANOVA, *F*_1,16_ = 39, *p* < 0.001). The P_i_‐supply and the species of N_i_‐source had no influence on the affinity (Supporting information Table S5).

**Figure 6:**
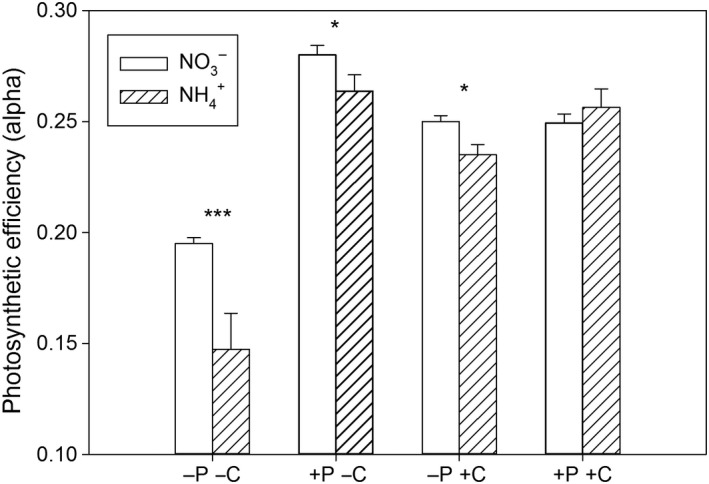
Mean and standard deviation of alpha which reflects the photosynthetic efficiency on the basis of electron transport rate (*n* = 3). Asterisks show differences between treatments varying in nitrogen sources (contrast analysis, * *p* < 0.05, *** *p* < 0.001), letters and signs of the *x*‐axis describe treatments: ‐P: P_i_‐limitation, +P: P_i_‐replete, ‐C: low CO_2_, +C: high CO_2_

The species of N_i_ played an important role under P_i_‐limited conditions in both CO_2_ conditions, as the photosynthetic efficiency (alpha) was lower when ${\text{NH}}_{4}^{+}$ was the N_i_‐source provided (Figure 6, contrast analysis, *p* < 0.05). Under P_i_‐replete conditions, significant influences of the N_i_‐source were only detected under low CO_2_ supply (contrast analysis, *p* < 0.05). Interactions were found between N_i_ and both P_i_ and CO_2_ (three‐way ANOVA, N_i_ × P_i_: *F*_1,15_ = 25.41, *p* < 0.001, N_i_ × CO_2_: *F*_1,15_ = 12.84, *p* < 0.01, Supporting information Table S5). The interaction between N_i_ and CO_2_ is represented by different intensities of an increased alpha. Thus, alpha was 32% higher in ${\text{NO}}_{3}^{-}$‐conditions relative to ${\text{NH}}_{4}^{+}$ under low CO_2_ and only 6% higher under high CO_2_. We observed the lowest values for alpha when P_i_ was limited, and CO_2_ was also low. Interestingly, the presence of only one of these nutrients in surplus enhanced the photosynthetic efficiency by about 50%, but phosphorus seemed to have a slightly stronger effect than CO_2_.

### 3.7 Carbon‐concentrating factor

The CCF is a factor indicating the presence of CCMs, which extent might be larger when a N_i_‐source is used that requires less metabolic energy to accumulate. Therefore, we expected a greater CCF in ${\text{NH}}_{4}^{+}$‐grown cells compared to ${\text{NO}}_{3}^{-}$‐grown cells, and yet we found no influence of the N_i_‐source, P_i_ and CO_2_ on the CCF and C_i_‐pool (three‐way ANOVA, all factors *p* > 0.22 Supporting information Table S6). Values of the CCF varied around 29 ± 10 (mean ± *SD*), when using the traditional method via tritium‐labeled water, to determine cell volume; or was 7.8 ± 4 (mean ± *SD*), when using the automatic cell counter for cell volume determination. The C_i_‐pool in the cells after 10 s was 2,535 ± 868 µM (mean ± *SD*). All values originated from three biological replicates per treatment, with *N* = 24; see Supporting information Table S6 for statistic details. CCF only varied depending on the method of calculation because the CASY estimated a larger cell volume. Therefore, the CCF calculated in the traditional way, by determining the cell volume with ^3^H‐H_2_O, was three times higher than the CCF calculated with the measured cell volume in the automatic cell counter (CASY).

### 3.8 Cell density

**Figure 7:**
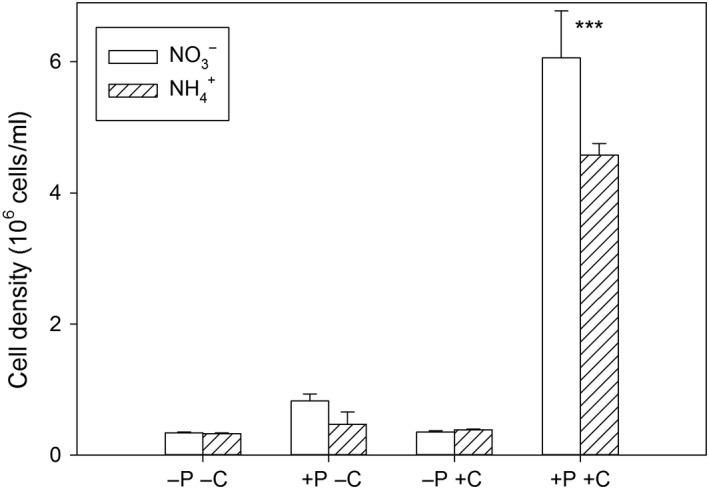
Mean and standard deviation of cell densities at the end of culturing (*n* = 3). Asterisks show differences between treatments differing in nitrogen sources (contrast analysis, *** *p* < 0.001), letters and signs of the *x*‐axis describe treatments: ‐P: P_i_‐limitation, +P: P_i_‐replete, ‐C: low CO_2_, +C: high CO_2_

The cell density is assumed to reflect the sum of all measured parameters, as it is the result of the population productivity. Cultures were grown at the same steady‐state growth rate of 0.2/d, which prevents the possibility to study growth rates for productivity. The three‐way ANOVA indicated that all factors have an impact on the cell density (N_i_ × P_i_ × CO_2_: *F*_1,16_ = 7.0, Figure 7, Supporting information Table S4). An influence of the N_i_‐source on cell density was only observed in cultures grown under P_i_‐replete and high CO_2_‐conditions (Figure 7, contrast analysis, *p* < 0.001). Under these conditions, the cell density was about 75% higher when ${\text{NO}}_{3}^{-}$ was provided, but as the ${\text{NH}}_{4}^{+}$‐cultures were N_i_‐limited, N_i_‐source had no effect on the cell density.

## 4 DISCUSSION

### 4.1 From nitrogen to amino acids

The relatively high glutamate content in cells grown with ${\text{NO}}_{3}^{-}$ probably reflects an accumulation of this amino acid due to a lower turnover of glutamate into other amino acids or proteins. This may be in line with our hypothesis that ${\text{NH}}_{4}^{+}$ allows a faster turnover of nitrogen into amino acids and through the subsequent metabolic pathways (e.g., into chl *a* synthesis; see below). In addition, under low CO_2_ conditions, ${\text{NO}}_{3}^{-}$‐grown cells had a higher total protein content than ${\text{NH}}_{4}^{+}$‐cultures (51 vs. 38 pg/cell, respectively; results not shown). These observations are consistent with a faster stimulation of amino acid synthesis when ${\text{NH}}_{4}^{+}$ was added to nitrogen‐starved cells of cyanobacteria, shown by Tapia, Ochoa de Alda, Llama, and Serra (1996). Opposing initial expectations, some other amino acids also reached higher concentrations in cells provided with ${\text{NO}}_{3}^{-}$ instead of ${\text{NH}}_{4}^{+}$. This phenomenon implies an effect of the N_i_‐source on downstream processes also; when, for example, the assembling of proteins proceeds slower. This hypothesis is supported by the detection of some higher amino acid contents in P_i_‐limited *C. acidophila*, than in P_i_‐replete cells as a higher production of amino acids would be intuitively expected under P_i_‐deficient, but N_i_‐replete conditions. Furthermore, high CO_2_ and P_i_‐limited conditions at the same time seemed to intensify the differences between different N_i_‐sources, as higher amounts of valine in ${\text{NO}}_{3}^{-}$‐cells were only seen under high CO_2_ supply. This might be an effect of a stronger P_i_‐limitation when high concentrations of CO_2_ are available as this was shown by a lower minimum cell quota for *C. acidophila* in a previous study (Spijkerman, Bissinger, Meister, & Gaedke, 2007).

### 4.2 Adaptations to acidic environment—NR activity

Contrary to our hypothesis that an acidophilic alga might have a preference for ammonium as an N_i_‐source, and possibly lacks NR activity as an adaptation to their environment, which consists of 91% ${\text{NH}}_{4}^{+}$ (Bissinger et al., 2000), we found NR activity in *C. acidophila.* We predominantly detected NR in ${\text{NO}}_{3}^{-}$‐grown cells, with high activities when ${\text{NO}}_{3}^{-}$ was provided under P_i_‐replete, low CO_2_ conditions: which has been known since at least 1969 for phytoplankton (by Eppley, Coatsworth, & Solorzano, 1969, who varied P_i_ conditions under low CO_2_ in cell cultures). The N_i_‐limitation in P_i_‐replete, high CO_2_, ${\text{NH}}_{4}^{+}$‐grown cells revealed that a N_i_‐limitation enhanced NR activity. The NR activity in this ${\text{NH}}_{4}^{+}$‐treatment was even higher than that in ${\text{NO}}_{3}^{-}$‐grown cells (Figure 3). Earlier, Kessler and Osterheld (1970) had also detected NR activity after ammonium was exhausted in cultures of two *Chlorella* strains. Probably, when ${\text{NH}}_{4}^{+}$ is exhausted, genes expressing NR (nit2) are activated (Fernandez & Galvan, 2007). Modern approaches suggest a role of the signaling molecule nitric oxide that functions as a signaling molecule in *Chlamydomonas *(Calatrava et al., 2017).

Interestingly, the NR activity in ${\text{NO}}_{3}^{-}$‐grown cells under low CO_2 _was higher in P_i_‐replete than in P_i_‐limited cells, supporting the assumption that an increased energy demand, in terms of ATP, for ${\text{NO}}_{3}^{-}$‐acquisition (Ruan & Giordano, 2017) was also present in *C. acidophila*.

### 4.3 Photosynthetic parameters—differences to neutrophiles/bicarbonate users

The higher ^14^C‐fixation rates in ${\text{NH}}_{4}^{+}$‐grown cells fits with the previously discussed faster turnover of nitrogen, expressed by a lower glutamate content in ${\text{NH}}_{4}^{+}$‐grown cells. This effect was only observed under a low CO_2_ condition, which is consistent with our hypothesis that effects from the N_i_‐source might be enhanced under low CO_2_ due to energy consuming CCMs (Raven & Beardall, 2014) or a decreased cellular ATP content (Raven et al., 2014). This was, however, not reflected in an enhanced biomass production in ${\text{NH}}_{4}^{+}$‐cultures, suggesting other metabolic costs.

We expected to find a more efficient photosynthesis when ${\text{NH}}_{4}^{+}$ was supplied instead of ${\text{NO}}_{3}^{-}$, but instead no effects were found. This, for example, is in contrast to a higher maximal photosynthetic rate in *Dunaliella salina* grown in ${\text{NH}}_{4}^{+}$ rather than ${\text{NO}}_{3}^{-}$ (Giordano, 1997), and a more efficient CCM in the same species (Giordano & Bowes, 1997). In *C. acidophila,* the CCF was similar among N_i_‐treatments, and also, no other indicators for more efficient CCMs in ${\text{NH}}_{4}^{+}$‐grown cells were found. Possibly, the different response originates from the large physiological differences in the CCM between the neutrophile, marine *Dunaliella salina* and our acidophile, freshwater *C. acidophila*. This difference is especially evident from the C_i_‐species supporting photosynthesis, as *D. salina *is able to use both bicarbonate and carbon dioxide, while *C. acidophila* is restricted to CO_2_ (Lachmann, Maberly, and Spijkerman 2016a,2016b). It has been proposed that ${\text{HCO}}_{3}^{-}$‐based CCMs requires more metabolic energy than CO_2_^‐^‐based ones (Raven et al., 2014), which we confirm here. Consequently, *D. salina* might be more dependent on active ${\text{HCO}}_{3}^{-}$‐uptake mechanisms in its CCM, and consequently photosynthetic parameters are more profoundly affected by N_i_‐source. Possibly, the metabolic advantages of ${\text{NH}}_{4}^{+}$‐grown cells for CO_2_ acquisition and photosynthesis in *C. acidophila* were compensated by enhanced proton extrusion necessities.

### 4.4 Cell density and chl *a*

An influence of the N_i_‐source on cell density was visible only in the treatment with the unexpected N_i_‐limitation, although the chl *a* content per cell was higher in ${\text{NH}}_{4}^{+}$‐grown cells than in ${\text{NO}}_{3}^{-}$‐grown cells under low CO_2_ and P_i_‐replete conditions. The latter was also shown for *D. salina *(Giordano, 1997). Because cell densities were independent of the N_i_‐source, other metabolic processes were enhanced in the ${\text{NH}}_{4}^{+}$‐grown cells (possibly related to proton extrusion). At P_i_‐limited conditions, the chl *a* content per cell did decrease slightly in cells grown on ${\text{NH}}_{4}^{+}$ compared to ${\text{NO}}_{3}^{-}$, under high CO_2_ conditions. Although we expected more pronounced differences due to a decreased energy budget in P_i_‐limited conditions (Lachmann et al., 2016a,b), the low chl *a* content might be close to a (minimum) threshold value, below which algae might face strong physiological restrictions, or below which the synthesis of chl *a* is prioritized for energy allocation (Spijkerman et al., 2007).

### 4.5 Methods to detect nutrient limitations

NIFT experiments revealed nutrient limitations and co‐limitations that remained hidden in (traditional) enrichment experiments. Enrichment experiments are often used because they are easier to conduct, as no special, expensive equipment is needed; however, they might fail to show a limitation in growth rate when the increase in biomass due to nutrient additions is low, for example, because cultures reach another limitation quickly. It was shown that NIFT experiments are also more suitable for detecting moderate nutrient limitations and co‐limitations, due to the rapid detection of the response (Spijkerman et al., 2016). We generally found a slight but consistently stronger response to additions of limiting nutrients in ${\text{NO}}_{3}^{-}$‐grown cells under low CO_2_, and in ${\text{NH}}_{4}^{+}$‐grown cells under high CO_2 _conditions. In contrast to results in enrichment experiments, we detected an unexpected (and unintended) N_i_‐limitation via NIFTs under a high supply of both P_i_ and CO_2_. Without this knowledge, we would have come to a false conclusion. Interestingly, the response to additions of more than one nutrient was much more complex when three nutrients were limiting (Figure 1c), suggesting complex physiological interactions among nutrient limitations (Koussoroplis, Pincebourde, & Wacker, 2017). It would be exciting to analyze this in further research, especially in relation to other physiological acclimations.

### 4.6 Conclusion and ecological implications

In conclusion, the effect of N_i_‐source on the studied physiological and metabolic traits of *C. acidophila* was very diverse and often influenced by C_i_‐ and/or P_i_‐limitation. Nitrate seemed to be the preferred N_i_‐source for photosynthesis and growth and led to more pronounced P_i_‐limitations under low CO_2_ conditions, emphasizing the enhanced energy requirement to assimilate this N_i_‐source. Our results suggest that the CO_2_‐user, *C. acidophila*, was little influenced in its C_i_‐acquisition by N_i_‐source, and that the CO_2_‐limitation seemed to be stronger under ${\text{NO}}_{3}^{-}$‐use. Possibly the use of ${\text{NH}}_{4}^{+}$ provides an additional proton stress for the cells that compensates for the metabolic advantages of this N_i_‐source (Giordano & Raven, 2014) as a higher mitochondrial activity is required (Weger et al., 1988).

None of our results suggested that *C. acidophila* developed special physiological adaptations to the higher concentrated N_i_‐source (i.e., ${\text{NH}}_{4}^{+}$) of their natural environment. Our species synthesized NR in the presence of ${\text{NO}}_{3}^{-}$: similar to neutrophiles (Chen et al., 2012; Li, Fingrut, & Maxwell, 2009). Local adaptation did not influence the N_i_‐source preference of this acidophile, whereas the green alga *Closterium aciculare* (Coesel, 1991), and most isolates from the acidotolerant *C. pitschmannii* (Pollio et al., 2005), were restricted to ammonium for their N_i_‐uptake. Such preferences are of interest for biofuel production, as lipid production in *Tetraselmis* sp. was higher when cells were grown with ${\text{NO}}_{3}^{-}$ than with ${\text{NH}}_{4}^{+}$. In general, effects of the N_i_‐source may be visible at the interactions among trophic levels. As *C. acidophila* is the most important photoautotroph, and the base of the food web in its lake of origin (Kamjunke, Gaedke, Tittel, Weithoff, & Bell, 2004), its physiological changes might have strong effects on its consumers and competitors.

## CONFLICT OF INTEREST

None Declared.

## AUTHORS CONTRIBUTION

The idea and design of experiments were conceived by SCL and ES. Laboratory work was conducted by SCL, ES (kinetic), and TMA (amino acids). SCL analyzed the data and wrote the manuscript. AW contributed to statistics of the study. SCL received comments to the written form of the manuscript by all co‐authors.

## Data Availability

Chemical and physiological data are accessible at Dryad (https://doi.org/10.5061/dryad.2k16k4b).
