# Supplementary material for: Nitrate or ammonium: Influences of nitrogen source on the physiology of a green alga
Source: Ecol Evol. 2019 Jan 10;9(3):1070–82. doi: 10.1002/ece3.4790 (PMC6374670; doi:10.1002/ece3.4790)
Supplement: Supplementary file 1 [file ECE3-9-1070-s001.docx]

**Supporting Information**

**Table S1:** Response of algae in optical density to the addition of listed nutrients. The relative difference in % of the response to addition of water was calculated. Values are the mean and standard deviation of three replicates given in parentheses. Italic numbers mark a certain nutrient limitation.

| **treatment** | **nutrient added** | **mean (response OD (%) relative to H_2_O)** |
| --- | --- | --- |
| NO3^-^, -P, -CO_2_ | P | *184 (80)* |
|  | N | -3 (9) |
|  | N & P | *170 (82)* |
|  | HCO_3_^-^ | -2 (3) |
| NH_4_^+^, -P, -CO_2_ | P | *250 (50)* |
|  | N | -3 (6) |
|  | N & P | *252 (73)* |
|  | HCO_3_^-^ | 3 (12) |
| NO3^-^, +P, -CO_2_ | P | 3 (10) |
|  | N | -1 (8) |
|  | N & P | -2 (17) |
|  | HCO_3_^-^ | -3 (5) |
| NH_4_^+^, +P, -CO_2_ | P | 7 (3) |
|  | N | 1 (4) |
|  | N & P | 7 (12) |
|  | HCO_3_^-^ | 3 (13) |
| NO3^-^, -P, +CO_2_ | P | *187 (54)* |
|  | N | 3 (7) |
|  | N & P | *184 (44)* |
|  | HCO_3_^-^ | -3 (10) |
| NH_4_^+^, -P, +CO_2_ | P | *239 (21)* |
|  | N | 3 (2) |
|  | N & P | *254 (27)* |
|  | HCO_3_^-^ | 2 (2) |
| NO3^-^, +P, +CO_2_ | P | 0 (0.5) |
|  | N | 0 (1.1) |
|  | N & P | 0 (0.3) |
|  | HCO_3_^-^ | -1 (2.4) |
| NH_4_^+^, +P, +CO_2_ | P | 2 (0) |
|  | N | 5 (2) |
|  | N & P | 4 (1) |
|  | HCO_3_^-^ | 3 (2) |

**Table S2:** Total particulate concentrations of N and P in cells of differently treated cultures, values are mean of triplicates ± standard deviation

| **treatment** | **mmol N/l** | **µmol P/l** |
| --- | --- | --- |
| NO_3_^-^, -P, -CO_2_ | 0.23 (0.04) | 1.3 (0.2) |
| NH_4_^+^, -P, -CO_2_ | 0.13 (0.00) | 1.3 (0.1) |
| NO_3_^-^_,_+P, -CO_2_ | 0.40 (0.05) | 51.2 (4.8) |
| NH_4_^+^_,_ +P, -CO_2_ | 0.31 (0.03) | 55.3 (1.7) |
| NO_3_^-^, -P, +CO_2_ | 0.23 (0.01) | 0.98 (0.05) |
| NH_4_^+^, -P, +CO_2_ | 0.17 (0.00) | 0.94 (0.08) |
| NO_3_^-^_,_+P, +CO_2_ | 1.94 (0.02) | 87.3 (2.4) |
| NH_4_^+^_,_ +P, +CO_2_ | 0.99 (0.04) | 89.4 (0.48) |

**Table S3:** Content of N and P per cell for different treatments, values are mean of triplicates ± standard deviation

| **treatment** | **fmol N/cell** | **fmol P/cell** |
| --- | --- | --- |
| NO_3_^-^, -P, -CO_2_ | 535 (93) | 3.1 (0.4) |
| NH_4_^+^, -P, -CO_2_ | 390 (5) | 3.8 (0.3) |
| NO_3_^-^_,_+P, -CO_2_ | 460 (42) | 58 (8) |
| NH_4_^+^_,_ +P, -CO_2_ | 456 (22) | 82 (4) |
| NO_3_^-^, -P, +CO_2_ | 598 (49) | 2.5 (0.4) |
| NH_4_^+^, -P, +CO_2_ | 426 (25) | 2.3 (0.1) |
| NO_3_^-^_,_+P, +CO_2_ | 346 (27) | 16 (2) |
| NH_4_^+^_,_ P+, +CO_2_ | 212 (3) | 19 (1) |

**Table S4:** Data of statistical analysis of cell density, chl a content per cell, NR activity and ^14^C-fixation rate. Degrees of freedom (df), the F-value and the statistical significance, p, are shown.

|  |  | **Cell density** | |  | **Chl *a* / cell** | |  | **NR activity** | |  | **^14^C-fix. rate** | |
| --- | --- | --- | --- | --- | --- | --- | --- | --- | --- | --- | --- | --- |
| **Factor** | **df** | **F** | **p** |  | **F** | **p** |  | **F** | **p** |  | **F** | **p** |
| **N** | 1, 16 | 16.75 | <0.001 |  | 49.42 | <0.001 |  | 103.7 | <0.001 |  | 4.96 | 0.041 |
| **P** | 1, 16 | 561.8 | <0.001 |  | 1568 | <0.001 |  | 114.4 | <0.001 |  | 59.19 | <0.001 |
| **CO_2_** | 1, 16 | 449.7 | <0.001 |  | 471.4 | <0.001 |  | 7.04 | 0.017 |  | 9.23 | 0.008 |
| **N x P** | 1, 16 | 17.5 | <0.001 |  | 42.8 | <0.001 |  | 9.36 | 0.007 |  | 2.975 | 0.104 |
| **N x CO_2_** | 1, 16 | 5.91 | 0.027 |  | 279.9 | <0.001 |  | 72.84 | <0.001 |  | 14.29 | 0.002 |
| **P x CO_2_** | 1, 16 | 435.9 | <0.001 |  | 38.08 | <0.001 |  | 27.71 | <0.001 |  | 18.15 | <0.001 |
| **N x P x CO_2_** | 1, 16 | 6.98 | 0.018 |  | 156.6 | <0.001 |  | 110.3 | <0.001 |  | 0.018 | 0.894 |

**Table S5:** Data of statistical analyses of kinetic parameters. Degrees of freedom (df), the F-value and the statistical significance, p, are shown.

|  |  | **V_max_** | |  | **K_0.5_(CO_2_)** | |  | **V_max_/K_0.5_** | |  | **alpha*** | |
| --- | --- | --- | --- | --- | --- | --- | --- | --- | --- | --- | --- | --- |
| **Factor** | **df** | **F** | **p** |  | **F** | **p** |  | **F** | **p** |  | **F** | **p** |
| **N** | 1, 16 | 6.05 | 0.003 |  | 2.44 | 0.138 |  | 0.16 | 0.699 |  | 20.72 | <0.001 |
| **P** | 1, 16 | 15.07 | 0.001 |  | 2.85 | 0.111 |  | 0.00 | 0.960 |  | 286.4 | <0.001 |
| **CO_2_** | 1, 16 | 17.31 | <0.001 |  | 72.02 | <0.001 |  | 38.89 | <0.001 |  | 76.78 | <0.001 |
| **N x P** | 1, 16 | 4.41 | 0.052 |  | 6.66 | 0.02 |  | 0.12 | 0.729 |  | 25.41 | <0.001 |
| **N x CO_2_** | 1, 16 | 4.53 | 0.049 |  | 2.31 | 0.148 |  | 0.16 | 0.691 |  | 12.8 | <0.001 |
| **P x CO_2_** | 1, 16 | 0.12 | 0.735 |  | 18.52 | <0.001 |  | 4.12 | 0.059 |  | 194.6 | <0.001 |
| **N x P x CO_2_** | 1, 16 | 0.002 | 0.965 |  | 5.08 | 0.039 |  | 0.07 | 0.799 |  | 0.514 | 0.484 |
|  |  |  |  |  |  |  |  |  |  |  | * df = 1,15 | |

**Table S6:** Data of statistical analyses of carbon-concentrating factors (CCF) and the internal inorganic carbon pool (C_i_-pool). Degrees of freedom (df), the F-value and the statistical significance, p, are shown.

|  |  | CCF (traditional) | |  | CFF (CASY) | |  | C_i_-pool | |
| --- | --- | --- | --- | --- | --- | --- | --- | --- | --- |
| Factor | df | F | p |  | F | p |  | F | p |
| N | 1, 16 | 0.51 | 0.485 |  | 0.82 | 0.380 |  | 1.09 | 0.312 |
| P | 1, 16 | 0.17 | 0.683 |  | 2.94 | 0.106 |  | 0.51 | 0.487 |
| CO_2_ | 1, 16 | 1.61 | 0.223 |  | 0.52 | 0.480 |  | 1.61 | 0.222 |
| N x P | 1, 16 | 0.20 | 0.658 |  | 2.40 | 0.141 |  | 0.09 | 0.773 |
| N x CO_2_ | 1, 16 | 0.05 | 0.824 |  | 0.00 | 0.995 |  | 0.08 | 0.786 |
| P x CO_2_ | 1, 16 | 0.02 | 0.899 |  | 0.30 | 0.589 |  | 0.09 | 0.764 |
| N x P x CO_2_ | 1, 16 | 0.20 | 0.664 |  | 1.03 | 0.326 |  | 0.01 | 0.913 |

**Table S7:** Data of statistical analyses of amino acids. Degrees of freedom (df), the F-value and the statistical significance, p, are shown

|  |  | **Glutamate** | | **Threonine** | | **Tyrosine** | | **Alanine** | | **Glycine** | | **Valine** | | **Isoleucine** | | **Leucine** | | **Phenylalanine** | |
| --- | --- | --- | --- | --- | --- | --- | --- | --- | --- | --- | --- | --- | --- | --- | --- | --- | --- | --- | --- |
| **Factor** | **df** | **F** | **p** | **F** | **p** | **F** | **p** | **F** | **p** | **F** | **p** | **F** | **p** | **F** | **p** | **F** | **p** | **F** | **p** |
| **N** | 1, 11 | 31,62 | <0.001 | 28.49 | <0.001 | 26.88 | <0.001 | 56.97 | <0.001 | 20.98 | <0.001 | 0.60 | 0.455 | 0.51 | 0.488 | 0.04 | 0.842 | 0.57 | 0.468 |
| **P** | 1, 11 | 0.91 | 0.362 | 13.84 | 0.01 | 1.61 | 0.230 | 55.65 | <0.001 | 72.85 | <0.001 | 7.76 | 0.018 | 8.65 | 0.013 | 16.26 | 0.002 | 1.61 | 0.230 |
| **CO_2_** | 1, 11 | 2.88 | 0.118 | 3.57 | 0.086 | 0.310 | 0.589 | 4.05 | 0.079 | 8.19 | 0.015 | 0.19 | 0.672 | 0.63 | 0.445 | 0.02 | 0.892 | 0.10 | 0.755 |
| **N x P** | 1, 11 | 0.94 | 0.353 | 5.89 | 0.033 | 0.692 | 0.23 | 41.41 | <0.001 | 89.51 | <0.001 | 2.93 | 0.115 | 0.78 | 0.397 | 0.06 | 0.817 | 0.26 | 0.620 |
| **N x CO_2_** | 1, 11 | 0.79 | 0.393 | 6..56 | 0.026 | 2.93 | 0.115 | 1.00 | 0.339 | 3.23 | 0.099 | 12.38 | 0.005 | 1.39 | 0.263 | 0.00 | 0.962 | 0.19 | 0.673 |
| **P x CO_2_** | 1, 11 | 1.77 | 0.210 | 2.63 | 0.133 | 0.494 | 0.497 | 9.62 | 0.010 | 22.44 | <0.001 | 3.58 | 0.085 | 4.44 | 0.059 | 1.49 | 0.248 | 0.67 | 0.430 |
| **N x P x CO_2_** | 1, 11 | 3.47 | 0.090 | 0.53 | 0.482 | 0.057 | 0.816 | 1.13 | 0.310 | 0.75 | 0.405 | 1.66 | 0.223 | 0.81 | 0.388 | 0.25 | 0.623 | 2.27 | 0.160 |
|  |  |  | |  | |  |  |  |  |  |  |  |  |  |  |  |  |  |  |
